# Two new species of *Erythromelana* Townsend, 1919 (Diptera: Tachinidae) from Area de Conservación Guanacaste in northwestern Costa Rica

**DOI:** 10.3897/BDJ.4.e7386

**Published:** 2016-04-19

**Authors:** AJ Fleming, D. Monty Wood, M. Alex Smith, Winnie Hallwachs, Daniel Janzen, Tanya Dapkey

**Affiliations:** ‡ Agriculture Agri-Food Canada, Ottawa, Canada; § Department of Integrative Biology and the Biodiversity Institute of Ontario, Guelph, Canada; | University of Pennsylvania, Philadelphia, United States of America

**Keywords:** tropical rain forest, tropical dry forest, cloud forest, parasitoid flies, host-specificity, caterpillars, ACG, Exoristinae, Blondeliini

## Abstract

**Background:**

We describe two new species in the genus *Erythromelana* Townsend, 1919 from Area de Conservación Guanacaste (ACG) in northwestern Costa Rica. Both species were reared from wild-caughtcaterpillars of *Eois* spp. (Lepidoptera: Geometridae). We provide a concise description of each species using morphology, life history, molecular data, and photographic documentation.

**New information:**

*Erythromelana jimmychevezi* Fleming & Wood **sp. nov.**

*Erythromelana glenriverai* Fleming & Wood **sp. nov.**

## Introduction

The tachinid genus *Erythromelana* Townsend, 1919 (Exoristinae: Blondeliini) is a small Neotropical genus in the tribe Blondeliini, occurring from southern Mexico to Bolivia and Brazil. Townsend originally described the genus based on one male and one female collected in Jaen province (Peru) and described as *E. jaena* Townsend. The genus remained untouched until Wood (1985) revised the entire tribe Blondeliini; in that work, Wood synonymized *Erythromelana* Townsend with *Minthomyia* Townsend, *Euptilodegeeria* Townsend and *Myiodoriops* Townsend, raising the total number of species within the genus to 6.

In 2013, an in-depth analysis and phylogeny of the genus *Erythromelana* was provided by Inclan and Stireman (2013), including the description of 11 new species. In addition to the new species descriptions, the authors revived two of the genera synonymized by Wood (1985) i.e. the monotypic genera *Euptilodegeeria* and *Myiodoriops*, and treated the nominal species *Erythromelana obscurifrons* Wulp as a *nomen dubium*, bringing the total number of recognized species in the genus to 14. Inclan and Stireman (2013) divided the genus into two species groups, the *E. jaena* group (*E. abdominalis* (Townsend), *E. curvifrons* Inclan, *E. ecuadoriana* Inclan, *E. eois* Inclan, *E. jaena* Townsend, *E. leptoforceps* Inclan and *E. nigrithorax* (Wulp)), and the *E. cryptica* group (*E. arciforceps* Inclan, *E. catarina* Inclan, *E. convexiforceps* Inclan, *E. cryptica* Inclan, *E. distincta* Inclan, *E. napensis* Inclan and *E. woodi* Inclan). Here, we build on this knowledge base by using CO1 (cox1 or cytochrome oxidase 1) gene sequences, or “DNA barcodes”, morphology, and life history, to describe two previously undescribed species from Costa Rica, which we place within the *E. cryptica* species-group. Both species are parasitoids of the small green *Eois* Hübner (Lepidoptera: Geometridae) caterpillars feeding on *Piper* L. spp. (Piperales: Piperaceae) within Area de Conservación Guanacaste (ACG) in northwestern Costa Rica. This paper forms part of a larger series of papers describing the parasitoid diversity of ACG (Smith et al. 2006, Smith et al. 2007, Janzen et al. 2009, Fleming et al. 2014a, Fleming et al. 2014b, Fleming et al. 2015c, Fleming et al. 2015a, Fleming et al. 2015b).

## Materials and methods

### Acronyms for depositories

CNC - Canadian National Collection of Insects, Arachnids and Nematodes, Ottawa, Canada

### Study area and rearing intensity

All flies and rearing information described here were obtained from the 35+ year–old ongoing inventory of the caterpillars, their food plants and their parasitoids of the dry forest, rain forest, cloud forest and intergrades of the 125,000+ ha terrestrial portion of Area de Conservación Guanacaste (ACG) in northwestern Costa Rica (Smith et al. 2006, Smith et al. 2007, Smith et al. 2008, Smith et al. 2009, Smith et al. 2012, Janzen et al. 2009, Janzen and Hallwachs 2011, Rodriguez et al. 2012, Fernandez-Triana et al. 2014, Fleming et al. 2014a). Parasitoid rearing methods are described at http://janzen.bio.upenn.edu/caterpillars/methodology/how/parasitoid_husbandry.htm.

### Imaging and dissections

Descriptions of new species discussed in this paper are deliberately brief, and only include characters commonly used in tachinid fly identification. The descriptions are complemented with color photos, in order to illustrate the readily observed inter-specific differences.

Photographs of the habitus and terminalia were taken using the methods outlined in Fleming et al. (2014a). In brief, raw image files were first processed with Adobe Photoshop CS6, and then digitally stacked to produce a final composite image using the Zerene Stacker software v1.04. Imaging of the male terminalia followed methods described in Fleming et al. 2014a. Dissections of the terminalia followed the methods described in O'Hara (1983).

The morphological terminology and measurements of body parts follow Inclan and Stireman (2013).

Wherever a specimen label has been examined, the information is presented using "/" to indicate the end of a label and the beginning of the next. Labels are presented from the top-most (closest to the specimen) to the bottom-most, with any additional comments given in square brackets" []".

### DNA barcoding

DNA barcodes (the standard 5’ region of the mitochondrial cytochrome oxidase 1 (CO1) gene) for all ACG inventory specimens were obtained using DNA extractions made from single legs, using a glass fiber protocol (Ivanova et al. 2006). Total genomic DNA was re-suspended in 30 μl of dH_2_O, and the 658-bp barcode region near the 5’ terminus of the CO1 gene was amplified using standard primers (LepF1–LepR1) and following established protocols (Smith et al. 2006, Smith et al. 2007, Smith et al. 2008). All information on the sequences associated with each individual specimen (including GenBank and BOLD accession numbers) can be retrieved from the Barcode of Life Data System (BOLD) (Ratnasingham and Hebert 2007) via the publicly available dataset dx.doi.org/10.5883/DS-ASERYTH. The neighbor-joining (NJ) tree (Saitou and Nei 1987) for the species of *Erythromelana* from ACG produced by BOLD is presented here as Suppl. material 1.

### Voucher specimen management

All caterpillars collected from the ACG efforts receive a unique voucher code in the format yy–SRNP–xxxxx. Any parasitoid emerging from a caterpillar receives the same voucher code, if/and when it is individually processed for DNA barcoding, it then receives a second voucher code unique to it, in the format DHJPARxxxxxxx. These voucher codes, assigned to the host and to any emerging parasitoids, can be looked up at http://janzen.bio.upenn.edu/caterpillars/database.lasso. To date, all voucher-coded tachinids have had one leg removed for attempted DNA barcoding at the Biodiversity Institute of Ontario (BIO) in Guelph, with all collateral data and all successful barcodes permanently and publicly deposited in the Barcode of Life Data System (BOLD, www.boldsystems.org) (Ratnasingham and Hebert 2007), and later migrated to GenBank as well. The inventory is dynamic and continually growing, with regular additions of new specimens. *Erythromelana* sequences can be found by searching for "*Erythromelana*" in BOLD. Each barcoded specimen has been assigned accession codes from the Barcode of Life Data System (BOLD) and GenBank.

The inventoried Tachinidae were collected under Costa Rican government research permits issued to DHJ and likewise exported under permit by DHJ from Costa Rica to Philadelphia, and then to their final depository in the CNC. Tachinid identifications for the inventory were done by DHJ in coordination with a) visual inspection by AJF and DMW, b) DNA barcoding by MAS, and c) correlation with host caterpillar identifications by DHJ and WH through the ACG inventory itself. Dates of capture of each specimen are the dates of eclosion of the flies, and not the dates of capture of the caterpillars since the eclosion date is much more representative of the time when that fly species is on the wing than is the time of capture of the caterpillar. The collector listed is the parataxonomist who collected the caterpillar, rather than the person who retrieved the newly eclosed fly and processed it by freezing, pinning, labeling and oven–drying. The biologies and parasitization rates of the flies will be the subject of later papers.

### Interim names of undescribed host species

Names of undescribed host species follow a standardized, interim naming system used for taxonomic units considered as distinct species and identified by DNA barcodes. The interim names are given in the format "*Eois* Janzen52", where the species epithet is composed of the name of the taxonomist who identified the species and a number. This prevents confusion with already described species while maintaining traceability of each undescribed species within the ACG project.

## Taxon treatments

### Erythromelana

Townsend, 1919

Erythromelana ***Erythromelana* Townsend, 1919**
Erythromelana Townsend, 1919a: 174. Type species: *Erythromelana jaena* Townsend, 1919a, by original designation. Other references: Guimarães (1971: 106); Wood (1985: 39–40); Wood & Zumbado (2011: 1403).
Minthomyia Townsend, 1919b: 564. Type species: *Minthomyia abdominalis* Townsend, 1919b, by original designation. Other references: Guimarães (1971: 41); Wood (1985: 39–40) (as synonym of *Erythromelana*).
Erythromelana Erythromelana jaena Townsend, 1919Townsend 1919: 174.

#### Description

*Erythromelana* can be distinguished from other Blondeliiniby the following combination of characters: **Head:** proclinate orbital setae absent in male, female with 2 setae; 1–2 pairs of reclinate outer orbital setae, lowermost outer orbital seta distinctly longer than uppermost frontal seta; ocellar setae ranging from absent to well developed; eyes haired, of variable density in different species; parafacial bare and extremely narrow, at narrowest point equal to or narrower than the basal width of the palpus; parafacial light gray in ground color, covered with silvery–gold pollinosity, and bare; fronto-orbital plate and vertex black in ground color, covered with a dull silver-gold pollinosity (appearing mostly black (Fig. 2b)), and with faint golden reflections visible only in lateral view (mostly on vertex); lower margin of face level with vibrissa; vibrissa positioned at extreme anteroventral corner of face; facial ridge with a few short hairs on lower third or less; arista black on basal 1/3–1/4, becoming light brownish to orange distally, elongate, and minutely pubescent, thickened only on basal 1/4; palpus light orange to yellow, sometimes darkened basally. **Thorax:** ground color shiny black; scutum silver pollinose presuturally, postsuturally a polished black in ground color; prosternum setose; postpronotum bearing 2 or, rarely, 3 setae; katepisternum with 2–3 setae; first postsutural supra-alar seta small or sometimes absent; apical scutellar setae lacking; wingvein R_4+5_ setulose at base and vein R_1_ setulose or bare; vein M smoothly curved at bend and ending at wing margin anterior to wing tip, separately from vein R_4+5_; legs ranging from entirely yellow to entirely black. **Abdomen:** ground color ranging from yellow-orange to black, without strong banding; mid-dorsal depression not extending to hind margin of T1+2; discal setae only present on T5. **Male terminalia:** sternite 5 with median cleft either U- or V-shaped; inner margin bearing minute setae; apical lobe rounded or pointed apically with either a single, long, well-developed seta, multiple well developed setae, or bare; pregonite curved anteriorly, with strong setae along posterior margin; postgonite short with curved apex; epiphallus small, usually difficult to see between the pregonites; surstylus with setulae on inner and outer surfaces, or completely bare; surstylus, in lateral view, varied in shape from almost straight to slightly concave on anterior or posterior margins, usually with a broad, rounded apex, occasionally truncate; surstylus and cercus usually subequal in length, sometimes cercus shorter than surstylus; in posterior view, cerci narrowed on apical 1/3.

#### Other species included

Based on synapomorphieswithin the male terminalia of *Erythromelana* described by Inclan and Stireman (2013), the two newly described species belong to the *E. cryptica* species-group and as such were compared to the other species belonging to that group.

*arciforceps* Inclan, 2013: 35. Holotype male (CNC). Type locality: Brazil, S.C., Nova Teutonia. Type label: Nova Teutonia S. C.-Brazil Nov. 1970 F. Plaumann/ HOLOTYPE *Erythromelana arciforceps* Inclan D.J/ DI412CA. Additional specimens examined: Brazil. [Examined by AJF.]

*catarina* Inclan, 2013: 32. Holotype male (CNC). Type locality: Brazil, S.C., Nova Teutonia. Type label: Nova Teutonia S. C.-Brazil Jun. 1970 F. Plaumann/ HOLOTYPE *Erythromelana catarina* Inclan D.J/ DI392CA. Additional specimens examined: Brazil. [Examined by AJF.]

*convexiforceps* Inclan, 2013: 33. Holotype male (CNC). Type locality: Mexico, Oaxaca, Suchistepec. Type label: Mexico, Oax 4.6 km Suchistepec 23.VII.1992 D.M. Wood 2150m/ HOLOTYPE *Erythromelana convexiforceps* Inclan D.J./ DI54CA. [Examined by AJF.]

*cryptica* Inclan, 2013: 29. Holotype male (CNC). Type locality: Venezuela, Aragua, Rancho Grande. Type label: VENEZUELA Aragua Rancho Grande 18-27.II.1971 G.&M. Wood 1100m/ HOLOTYPE *Erythromelana cryptica* Inclan D.J./ DI477CA. Additional specimens examined: Venezuela, Bolivia, Mexico and Ecuador. [Examined by AJF.]

*distincta* Inclan, 2013: 39. Holotype male (CNC). Type locality: Venezuela, Aragua, Rancho Grande. Type label: VENEZUELA Aragua 11 km Rancho Grande 25.II.1971 G.&M. Wood/ HOLOTYPE *Erythromelana distincta* Inclan D.J./ DI280CA. Additional specimens examined: Brazil and Venezuela. [Examined by AJF.]

*napensis* Inclan, 2013: 37. Holotype male (CNC). Type locality: Ecuador, Napo prov., Yanayacu Biological Station. Type label: ECUADOR: Napo Prov. Yanayacu Biological Station S00°35.9', W77°53.4', 2163 m REARED October 2005 8135/ HOLOTYPE *Erythromelana napensis* Inclan D.J. [Examined by AJF.]

*woodi* Inclan, 2013: 42. Holotype male (CNC). Type locality: Costa Rica, Puntarenas, Monteverde. Type label: COSTA RICA Pnts Monteverde 28.VIII.1993 D.M. Wood 1842m/ HOLOTYPE *Erythromelana woodi* Inclan D.J./ DI208MW. Additional specimens examined: Costa Rica, Bolivia, Mexico and Ecuador. [Examined by AJF.]

### Erythromelana jimmychevezi

Fleming & Wood sp. n.

urn:lsid:zoobank.org:act:9DD705C8-E949-47DE-B8EB-9A7A979EBD00

#### Materials

- **Type status:** Holotype. **Occurrence:** occurrenceDetails: http://janzen.sas.upenn.edu; catalogNumber: DHJPAR0038684; recordedBy: D.H. Janzen & W. Hallwachs, Dinia Martinez; individualID: DHJPAR0038684; individualCount: 1; sex: male; lifeStage: adult; preparations: pinned; otherCatalogNumbers: 10-SRNP-70648,ASHYD2257-10,BOLD:AAL5493; **Taxon:** scientificName: Erythromelana jimmychevezi; phylum: Arthropoda; class: Insecta; order: Diptera; family: Tachinidae; genus: Erythromelana; specificEpithet: jimmychevezi; scientificNameAuthorship: Fleming & Wood; **Location:** continent: Central America; country: Costa Rica; countryCode: CR; stateProvince: Guanacaste; county: Sector Pitilla; locality: Area de Conservacion Guanacaste; verbatimLocality: Estacion Quica; verbatimElevation: 470; verbatimLatitude: 10.997; verbatimLongitude: -85.397; verbatimCoordinateSystem: Decimal; decimalLatitude: 10.997; decimalLongitude: -85.397; **Identification:** identifiedBy: AJ Fleming; dateIdentified: 2015; **Event:** samplingProtocol: Reared from a Geometrid moth larva, *Eois* Janzen52; verbatimEventDate: 27-Feb-2010; **Record Level:** language: en; institutionCode: CNC; collectionCode: Insects; basisOfRecord: Pinned Specimen
- **Type status:** Paratype. **Occurrence:** occurrenceDetails: http://janzen.sas.upenn.edu; catalogNumber: DHJPAR0037257; recordedBy: D.H. Janzen & W. Hallwachs, Manuel Rios; individualID: DHJPAR0037257; individualCount: 1; sex: female; lifeStage: adult; preparations: pinned; otherCatalogNumbers: 09-SRNP-33247,ASHYC4002-10,BOLD:AAL5493; **Taxon:** scientificName: Erythromelana jimmychevezi; phylum: Arthropoda; class: Insecta; order: Diptera; family: Tachinidae; genus: Erythromelana; specificEpithet: jimmychevezi; scientificNameAuthorship: Fleming & Wood; **Location:** continent: Central America; country: Costa Rica; countryCode: CR; stateProvince: Guanacaste; county: Sector Pitilla; locality: Area de Conservacion Guanacaste; verbatimLocality: Estacion Pitilla; verbatimElevation: 675; verbatimLatitude: 10.989; verbatimLongitude: -85.426; verbatimCoordinateSystem: Decimal; decimalLatitude: 10.989; decimalLongitude: -85.426; **Identification:** identifiedBy: AJ Fleming; dateIdentified: 2015; **Event:** samplingProtocol: Reared from a Geometrid moth larva, *Eois* Janzen52; verbatimEventDate: 08-Dec-2009; **Record Level:** language: en; institutionCode: CNC; collectionCode: Insects; basisOfRecord: Pinned Specimen
- **Type status:** Paratype. **Occurrence:** occurrenceDetails: http://janzen.sas.upenn.edu; catalogNumber: DHJPAR0040781; recordedBy: D.H. Janzen & W. Hallwachs, Dinia Martinez; individualID: DHJPAR0040781; individualCount: 1; sex: male; lifeStage: adult; preparations: pinned, with dissected terminalia in glycerine microvial on pin; otherCatalogNumbers: 10-SRNP-70701,ASHYE2917-11,BOLD:AAL5493; **Taxon:** scientificName: Erythromelana jimmychevezi; phylum: Arthropoda; class: Insecta; order: Diptera; family: Tachinidae; genus: Erythromelana; specificEpithet: jimmychevezi; scientificNameAuthorship: Fleming & Wood, 2016; **Location:** continent: Central America; country: Costa Rica; countryCode: CR; stateProvince: Guanacaste; county: Sector Pitilla; locality: Area de Conservacion Guanacaste; verbatimLocality: Estacion Quica; verbatimElevation: 470; verbatimLatitude: 10.997; verbatimLongitude: -85.397; verbatimCoordinateSystem: Decimal; decimalLatitude: 10.997; decimalLongitude: -85.397; **Identification:** identifiedBy: AJ Fleming; dateIdentified: 2015; **Event:** samplingProtocol: Reared from a Geometrid moth larva, *Eois* Janzen52; verbatimEventDate: 02-Mar-2010; **Record Level:** language: en; institutionCode: CNC; collectionCode: Insects; basisOfRecord: Pinned Specimen
- **Type status:** Paratype. **Occurrence:** occurrenceDetails: http://janzen.sas.upenn.edu; catalogNumber: DHJPAR0040786; recordedBy: D.H. Janzen & W. Hallwachs, Dinia Martinez; individualID: DHJPAR0040786; individualCount: 1; sex: female; lifeStage: adult; preparations: pinned; otherCatalogNumbers: 10-SRNP-70698,ASHYE2922-11,BOLD:AAL5493; **Taxon:** scientificName: Erythromelana jimmychevezi; phylum: Arthropoda; class: Insecta; order: Diptera; family: Tachinidae; genus: Erythromelana; specificEpithet: jimmychevezi; scientificNameAuthorship: Fleming & Wood, 2016; **Location:** continent: Central America; country: Costa Rica; countryCode: CR; stateProvince: Guanacaste; county: Sector Pitilla; locality: Area de Conservacion Guanacaste; verbatimLocality: Estacion Quica; verbatimElevation: 470; verbatimLatitude: 10.997; verbatimLongitude: -85.397; verbatimCoordinateSystem: Decimal; decimalLatitude: 10.997; decimalLongitude: -85.397; **Identification:** identifiedBy: AJ Fleming; dateIdentified: 2015; **Event:** samplingProtocol: Reared from a Geometrid moth larva, *Eois* Janzen52; verbatimEventDate: 02-Mar-2010; **Record Level:** language: en; institutionCode: CNC; collectionCode: Insects; basisOfRecord: Pinned Specimen

#### Description

Described from 2 males and 2 females. Length: male = 6mm; female = 5–6mm.

**Head:** (Fig. 2b, e) eye haired, with long ommatrichia about as long as combined width of 4–5 eye facets; eye approximately 0.9x head height; vertex 0.12–0.14x head width in male, 0.21–0.23x head width in female; frontal vitta 0.33–0.35x vertex width in male, 0.32–0.34x vertex width in female; first flagellomere black, reaching facial margin; fronto-orbital plate with 2 reclinate inner orbital setae, the lowermost large and well developed in contrast to the uppermost, which is small or reduced, almost hair-like (especially in the female); ocellar setae proclinate; facial ridge bare; palpus yellowish, with dark umber base, distally haired and almost uniform in width.

**Thorax:** (Fig. 2a, c, d, f) dorsal vittae faintly visible as slightly darker stripes, the inner pair longer and thinner than the outer pair; postpronotum with 2 setae, often with one additional small seta; three postsutural supra-alar setae; katepisternum with 3 setae, ventromedial seta less than 1/3 the length of anteroventral seta; scutellar discal setae absent; legs black; wing smoky gray, vein R_1_ dorsally bare, vein R_4+5_ dorsally with 3–4 setulae at base.

**Abdomen:** dorsal surface of abdomen mostly black in ground color; ventral margins of T1+2 and T3 yellow in ground color, the yellow extending to 3/4 of lateral surface of these tergites; T4 yellow in ground color on 1/3–1/2 of lateral surface; in dorsal view, both T3 and T4 black in ground color medially and yellow laterally; ground color of T5 entirely black; transverse bands of sparse white pollinosisty present on anterior 1/3–1/4 of T3 and T4 and on anterior 2/3 of T5; one pair of median marginal setae on T1+2 and T3; T4 and T5 with a full row of marginal setae; mid-dorsal depression of T1+2 not reaching median marginal setae.

**Male terminalia:** (Fig. 3) sternite 5 with median cleft V-shaped; apical lobes broadly pointed, each with 2–3 long, well-developed setae, the longest seta at least 2x as long as second longest; surstylus with small hairs on inner and outer surfaces; surstylus, in lateral view, almost straight on basal 1/2 and convex on apical 1/2 of anterior margin, and very slightly concave along posterior margin; surstylus and cercus almost equal in length in lateral view (Fig. 3b); cercus, in lateral view, bent, digitate, not strongly concave on anterior surface, very slightly concave on postero-apical margin, and with a rounded tip; in posterior view, cerci narrowed inapical 1/3, length of upper lobes almost equal to medial section and longer than the apical cleft; apical cleft well defined with rounded tips directed slightly medially, basal section 2/3 the length of apical section.

#### Diagnosis

This species is included in the *E. cryptica* species group (Inclan and Stireman 2013) because of its morphological similarity to other members of the group. *Erythromelana jimmychevezi* can bedistinguished from *E. arciforceps* by the following character states: light brown wings; multiple, strong apical setae on the lobes of sternite 5; more pointed appearance to the apices of the lobes of sternite 5. It is worth noting that *E. jimmychevezi* may overlap in range with *E. arciforceps*, which has been collected from as far north as Monteverde, Costa Rica.

#### Etymology

*Erythromelana jimmychevezi* is named in recognition of Jimmy Chévez Elizondo for his contributions to the accounting team for Area de Conservación Guanacaste, the forest this fly lives in.

#### Distribution

Costa Rica, ACG, Guanacaste Prov., rain forest, 470–675 m elevation.

#### Ecology

A parasitoid of *Eois* Janzen52 (Geometridae), which feeds on *Piper auritum* (Piperaceae).

### Erythromelana glenriverai

Fleming & Wood sp. n.

urn:lsid:zoobank.org:act:2EEF889B-CA19-4970-B20E-1283E958D027

#### Materials

- **Type status:** Holotype. **Occurrence:** occurrenceDetails: http://janzen.sas.upenn.edu; catalogNumber: DHJPAR0038680; recordedBy: D.H. Janzen & W. Hallwachs, Calixto Moraga; individualID: DHJPAR0038680; individualCount: 1; sex: male; lifeStage: adult; preparations: pinned; otherCatalogNumbers: 10-SRNP-30496,ASHYD2253-10,BOLD:AAL4742; **Taxon:** scientificName: Erythromelana glenriverai; phylum: Arthropoda; class: Insecta; order: Diptera; family: Tachinidae; genus: Erythromelana; specificEpithet: glenriverai; scientificNameAuthorship: Fleming & Wood; **Location:** continent: Central America; country: Costa Rica; countryCode: CR; stateProvince: Guanacaste; county: Sector Pitilla; locality: Area de Conservacion Guanacaste; verbatimLocality: Sendero Montecele; verbatimElevation: 680; verbatimLatitude: 10.973; verbatimLongitude: -85.421; verbatimCoordinateSystem: Decimal; decimalLatitude: 10.973; decimalLongitude: -85.421; **Identification:** identifiedBy: AJ Fleming; dateIdentified: 2015; **Event:** samplingProtocol: Reared from a Geometrid moth larva, *Eois* Janzen04; verbatimEventDate: 11-Mar-2010; **Record Level:** language: en; institutionCode: CNC; collectionCode: Insects; basisOfRecord: Pinned Specimen
- **Type status:** Paratype. **Occurrence:** occurrenceDetails: http://janzen.sas.upenn.edu; catalogNumber: DHJPAR0034607; recordedBy: D.H. Janzen & W. Hallwachs, Calixto Moraga; individualID: DHJPAR0034607; individualCount: 1; sex: male; lifeStage: adult; preparations: pinned; otherCatalogNumbers: 09-SRNP-31595,ASHYC1259-09,BOLD:AAL4742; **Taxon:** scientificName: Erythromelana glenriverai; phylum: Arthropoda; class: Insecta; order: Diptera; family: Tachinidae; genus: Erythromelana; specificEpithet: glenriverai; scientificNameAuthorship: Fleming & Wood; **Location:** continent: Central America; country: Costa Rica; countryCode: CR; stateProvince: Guanacaste; county: Sector Pitilla; locality: Area de Conservacion Guanacaste; verbatimLocality: Sendero Memos; verbatimElevation: 740; verbatimLatitude: 10.982; verbatimLongitude: -85.428; verbatimCoordinateSystem: Decimal; decimalLatitude: 10.982; decimalLongitude: -85.428; **Identification:** identifiedBy: AJ Fleming; dateIdentified: 2015; **Event:** samplingProtocol: Reared from a Geometrid moth larva, *Eois* Janzen04; verbatimEventDate: 03-Jun-2009; **Record Level:** language: en; institutionCode: CNC; collectionCode: Insects; basisOfRecord: Pinned Specimen
- **Type status:** Paratype. **Occurrence:** occurrenceDetails: http://janzen.sas.upenn.edu; catalogNumber: DHJPAR0037426; recordedBy: D.H. Janzen & W. Hallwachs, Calixto Moraga; individualID: DHJPAR0037426; individualCount: 1; sex: female; lifeStage: adult; preparations: pinned; otherCatalogNumbers: 10-SRNP-30070,ASHYC4171-10,BOLD:AAL4742; **Taxon:** scientificName: Erythromelana glenriverai; phylum: Arthropoda; class: Insecta; order: Diptera; family: Tachinidae; genus: Erythromelana; specificEpithet: glenriverai; scientificNameAuthorship: Fleming & Wood; **Location:** continent: Central America; country: Costa Rica; countryCode: CR; stateProvince: Guanacaste; county: Sector Pitilla; locality: Area de Conservacion Guanacaste; verbatimLocality: Sendero Memos; verbatimElevation: 740; verbatimLatitude: 10.982; verbatimLongitude: -85.428; verbatimCoordinateSystem: Decimal; decimalLatitude: 10.982; decimalLongitude: -85.428; **Identification:** identifiedBy: AJ Fleming; dateIdentified: 2015; **Event:** samplingProtocol: Reared from a Geometrid moth larva, *Eois* Janzen04; verbatimEventDate: 27-Jan-2010; **Record Level:** language: en; institutionCode: CNC; collectionCode: Insects; basisOfRecord: Pinned Specimen
- **Type status:** Paratype. **Occurrence:** occurrenceDetails: http://janzen.sas.upenn.edu; catalogNumber: DHJPAR0042703; recordedBy: D.H. Janzen & W. Hallwachs, Petrona Rios; individualID: DHJPAR0042703; individualCount: 1; sex: female; lifeStage: adult; preparations: pinned; otherCatalogNumbers: 11-SRNP-69794,ASHYH461-11,BOLD:AAL4742; **Taxon:** scientificName: Erythromelana glenriverai; phylum: Arthropoda; class: Insecta; order: Diptera; family: Tachinidae; genus: Erythromelana; specificEpithet: glenriverai; scientificNameAuthorship: Fleming & Wood; **Location:** continent: Central America; country: Costa Rica; countryCode: CR; stateProvince: Alajuela; county: Sector Rincon Rain Forest; locality: Area de Conservacion Guanacaste; verbatimLocality: Jacobo; verbatimElevation: 461; verbatimLatitude: 10.941; verbatimLongitude: -85.318; verbatimCoordinateSystem: Decimal; decimalLatitude: 10.941; decimalLongitude: -85.318; **Identification:** identifiedBy: AJ Fleming; dateIdentified: 2015; **Event:** samplingProtocol: Reared from a Geometrid moth larva, *Eois dibapha*; verbatimEventDate: 11-Apr-2011; **Record Level:** language: en; institutionCode: CNC; collectionCode: Insects; basisOfRecord: Pinned Specimen
- **Type status:** Paratype. **Occurrence:** occurrenceDetails: http://janzen.sas.upenn.edu; catalogNumber: DHJPAR0044885; recordedBy: D.H. Janzen & W. Hallwachs, Anabelle Cordoba; individualID: DHJPAR0044885; individualCount: 1; sex: female; lifeStage: adult; preparations: pinned; otherCatalogNumbers: 11-SRNP-43251,ACGAZ109-11,BOLD:AAL4742; **Taxon:** scientificName: Erythromelana glenriverai; phylum: Arthropoda; class: Insecta; order: Diptera; family: Tachinidae; genus: Erythromelana; specificEpithet: glenriverai; scientificNameAuthorship: Fleming & Wood; **Location:** continent: Central America; country: Costa Rica; countryCode: CR; stateProvince: Guanacaste; county: Sector Rincon Rain Forest; locality: Area de Conservacion Guanacaste; verbatimLocality: Sendero Rincon; verbatimElevation: 430; verbatimLatitude: 10.896; verbatimLongitude: -85.278; verbatimCoordinateSystem: Decimal; decimalLatitude: 10.896; decimalLongitude: -85.278; **Identification:** identifiedBy: AJ Fleming; dateIdentified: 2015; **Event:** samplingProtocol: Reared from a Geometrid moth larva, *Eois* Janzen236; verbatimEventDate: 24-Jul-2011; **Record Level:** language: en; institutionCode: CNC; collectionCode: Insects; basisOfRecord: Pinned Specimen
- **Type status:** Paratype. **Occurrence:** occurrenceDetails: http://janzen.sas.upenn.edu; catalogNumber: DHJPAR0053260; recordedBy: D.H. Janzen & W. Hallwachs, Calixto Moraga; individualID: DHJPAR0053260; individualCount: 1; sex: male; lifeStage: adult; preparations: pinned, with dissected terminalia in glycerine microvial on pin; otherCatalogNumbers: 13-SRNP-31078,ASHYM2614-13,BOLD:AAL4742; **Taxon:** scientificName: Erythromelana glenriverai; phylum: Arthropoda; class: Insecta; order: Diptera; family: Tachinidae; genus: Erythromelana; specificEpithet: glenriverai; scientificNameAuthorship: Fleming & Wood; **Location:** continent: Central America; country: Costa Rica; countryCode: CR; stateProvince: Guanacaste; county: Sector Pitilla; locality: Area de Conservacion Guanacaste; verbatimLocality: Sendero Evangelista; verbatimCoordinateSystem: Decimal; **Identification:** identifiedBy: AJ Fleming; dateIdentified: 2015; **Event:** samplingProtocol: Reared from a Geometrid moth larva, *Eois* Janzen49; verbatimEventDate: 02-Sep-2013; **Record Level:** language: en; institutionCode: CNC; collectionCode: Insects; basisOfRecord: Pinned Specimen
- **Type status:** Paratype. **Occurrence:** occurrenceDetails: http://janzen.sas.upenn.edu; catalogNumber: DHJPAR0053261; recordedBy: D.H. Janzen & W. Hallwachs, Calixto Moraga; individualID: DHJPAR0053261; individualCount: 1; sex: male; lifeStage: adult; preparations: pinned; otherCatalogNumbers: 13-SRNP-31079,ASHYM2615-13,BOLD:AAL4742; **Taxon:** scientificName: Erythromelana glenriverai; phylum: Arthropoda; class: Insecta; order: Diptera; family: Tachinidae; genus: Erythromelana; specificEpithet: glenriverai; scientificNameAuthorship: Fleming & Wood; **Location:** continent: Central America; country: Costa Rica; countryCode: CR; stateProvince: Guanacaste; county: Sector Pitilla; locality: Area de Conservacion Guanacaste; verbatimLocality: Sendero Evangelista; verbatimCoordinateSystem: Decimal; **Identification:** identifiedBy: AJ Fleming; dateIdentified: 2015; **Event:** samplingProtocol: Reared from a Geometrid moth larva, *Eois* Janzen49; verbatimEventDate: 02-Sep-2013; **Record Level:** language: en; institutionCode: CNC; collectionCode: Insects; basisOfRecord: Pinned Specimen
- **Type status:** Paratype. **Occurrence:** occurrenceDetails: http://janzen.sas.upenn.edu; catalogNumber: DHJPAR0053264; recordedBy: D.H. Janzen & W. Hallwachs, Calixto Moraga; individualID: DHJPAR0053264; individualCount: 1; sex: female; lifeStage: adult; preparations: pinned; otherCatalogNumbers: 13-SRNP-31082,ASHYM2618-13,BOLD:AAL4742; **Taxon:** scientificName: Erythromelana glenriverai; phylum: Arthropoda; class: Insecta; order: Diptera; family: Tachinidae; genus: Erythromelana; specificEpithet: glenriverai; scientificNameAuthorship: Fleming & Wood; **Location:** continent: Central America; country: Costa Rica; countryCode: CR; stateProvince: Guanacaste; county: Sector Pitilla; locality: Area de Conservacion Guanacaste; verbatimLocality: Sendero Evangelista; verbatimCoordinateSystem: Decimal; **Identification:** identifiedBy: AJ Fleming; dateIdentified: 2015; **Event:** samplingProtocol: Reared from a Geometrid moth larva, *Eois* Janzen49; verbatimEventDate: 02-Sep-2013; **Record Level:** language: en; institutionCode: CNC; collectionCode: Insects; basisOfRecord: Pinned Specimen

#### Description

Described from 4 males and 4 females. Length: male = 6–7mm; female = 5–6mm .

**Head:** (Fig. 4b, e) eye haired, with long ommatrichia about as long as combined width of 4–5 eye facets; eye approximately 0.9x head height; vertex width 0.13–0.15x head width in male, 0.22–0.24x head width in female; frontal vitta width 0.43–0.46x vertex width in male, 0.32–0.34x vertex width in female; first flagellomere black, reaching facial margin; fronto-orbital plate with 2 reclinate inner orbital setae of subequal length in male, whereas in the female the uppermost is large and well developed, 2x the length of the lowermost; ocellar setae small, poorly developed and proclinate; facial ridge bare; palpus yellowish, with dark brown-yellow base; distally haired; almost uniform in width.

**Thorax:** (Fig. 4a, c, d, f) dorsal vittae faintly visible presuturally as slightly darker stripes, virtually invisible postsuturally; postpronotum with 2 setae, usually with one additional small seta; two postsutural supra-alar setae (rarely only 1); katepisternum with 3 setae, ventromedial seta poorly developed, almost hair-like; scutellar discal setae absent; legs black; wing smoky gray, vein R_1_ dorsally bare, vein R_4+5_ dorsally with 3–4 setulae at base in male, and 5–6 setulae at base in female.

**Abdomen:** ground color of dorsal surface mostly black; T1+2 all black in ground color in dorsal view, and of yellow ground color ventrally in lateral view; T3 mostly yellow on anterior 3/4, black posteriorly; coloration of T3, in dorsal view, appearing as a black triangle on a yellow background; T4 yellow in ground color on 1/3–1/2 of lateral surface, appearing as entirely black in dorsal view; T5 entirely black; transverse bands of sparse white pollinosity present on anterior 1/3–1/4 of T3 and T4, and on anterior 2/3 of T5; one pair of median marginal setae on T1+2 and T3; T4 and T5 with a full row of marginal setae; mid-dorsal depression of T1+2 not reaching median marginal setae.

**Male terminalia:** (Fig. 5) sternite 5 with median cleft V-shaped, apical lobes bearing rounded apices, each with 1 long, well-developed seta, the longest seta at least 4x as long as the second longest; anterior margin of basal plate slightly concave; surstylus with small hairs on inner and outer surfaces; in lateral view, surstylus almost straight on basal 1/2 and convex on apical 1/2 of anterior margin, and very slightly concave along posterior margin; in lateral view, surstylus and cercus almost equal in length; cercus in lateral view bent, digitate, strongly concave on anterior surface, very slightly concave on postero-apical margin, and with a rounded tip; cercus truncate in lateral view, appearing like a slightly bent thumb; in posterior view, cerci narrowed on apical 1/3, length of upper lobes almost equal to medial section and longer than the apical cleft, basal section 1/3 longer than apical section; apical cleft well defined, with rounded tips directed medially.

#### Diagnosis

**Figure 1.**
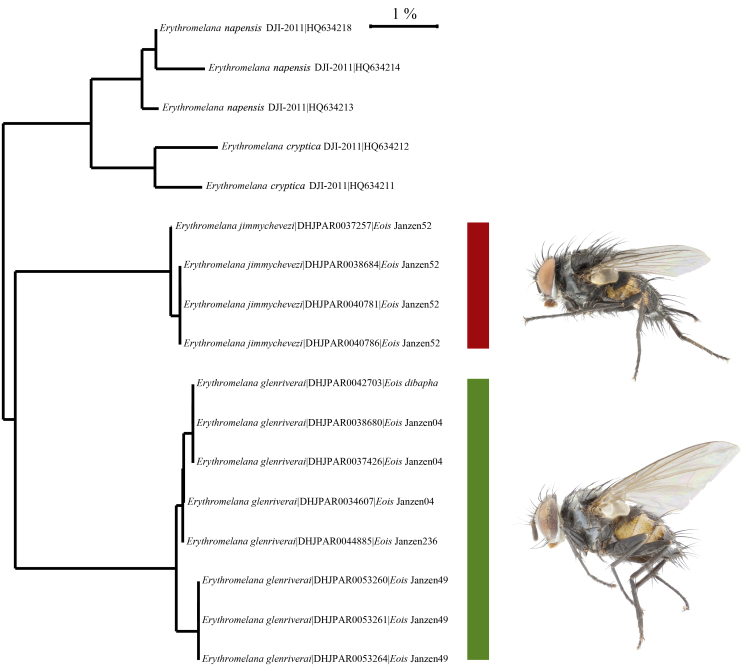
Neighbor-Joining (NJ – Saitou and Nei 1987) tree comparing the two species of *Erythromelana* present in ACG and the GenBank sequences of *Erythromelana cryptica* and *E. napensis*. Tree based on Kimura 2-parameter distances (K2P – Kimura 1980) made using MEGA6 (Tamura et al. 2013). Tip labels include species name, individual specimen ID (accession numbers for the Inclan and Stireman (2013) specimens, and voucher codes for the ACG specimens) and, for the two new species described herein, host species and the image of a male in lateral view. This phenogram demonstrates the low intra-specific and high inter-specific variation in CO1 barcode sequences.

This species is included in the *E. cryptica* species group (Inclan and Stireman 2013) because of its morphological similarity to other members of the group. *Erythromelana glenriverai* can bedistinguished from *E. cryptica* by the following characters: consistently having only two postsutural supra-alar setae; a less developed medial katepisternal seta; a more spatulate surstylus (visible in both lateral and posterior views); and a more curved, narrower anterior section of cercus (in posterior view). Additionally, the posterior section of the cercus in *E. glenriverai* is almost equal in length to the length of both the anterior and medial sections combined. *Erythromelana cryptica* was one of the few species for which a barcode was available among the previously described species; its sequence was also different from that of *E. glenriverai* (Fig. 1) .

#### Etymology

*Erythromelana glenriverai* is named in recognition of Glen Rivera Chaves for his contributions to the accounting team for Area de Conservación Guanacaste, the forest this fly lives in.

#### Distribution

Costa Rica, ACG, Alajuela and Guanacaste Provs., rain forest, 430–740m elevation.

#### Ecology

A parasitoid of *Eois* Janzen49, *E.* Janzen04, *E.* Janzen236 and *E. dibapha* (Schaus) (Geometridae), which feed on four species of rain forest Piperaceae.

## Analysis

The DNA barcode sequences recovered from the new *Erythromelana* species from ACG display the characteristic strong AT bias of insect mitochondrial DNA (mean percent GC content 32.89%, SE 0.08) and no insertions or deletions. Within-species variation was low (mean distance of 0.28%) compared to between-species variation (mean distance 5.18%). All values of DNA barcode variation were calculated within BOLD and can be re-calculated in the future as more specimens are recovered from the ACG inventory and added to the DNA library. Fig. 1 presents a neighbor–joining tree for the *Erythromelana* specimens reared and DNA barcoded by this inventory to date.

## Supplementary Material

Supplementary material 1 NJ Tree Erythromelana (ACG) Data type: Neighbor-joining tree
File: oo_63489.pdf Fleming et. al 2015

XML Treatment for Erythromelana

XML Treatment for Erythromelana jimmychevezi

XML Treatment for Erythromelana glenriverai

## Figures and Tables

**Figure 2a.**
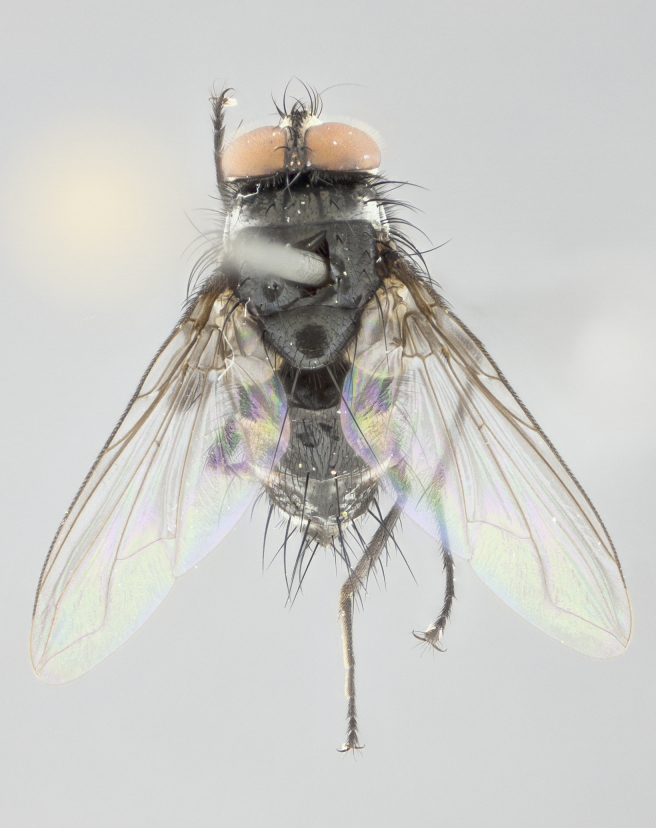
habitus in dorsal view

**Figure 2b.**
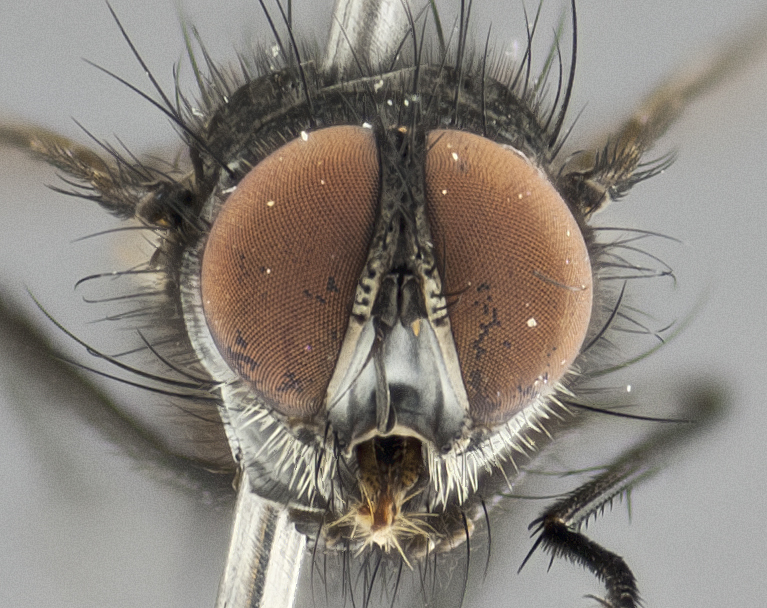
head in frontal view

**Figure 2c.**
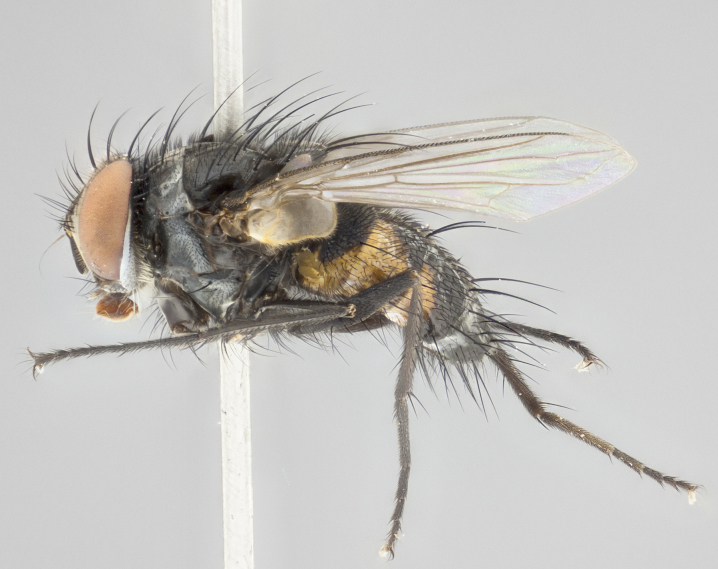
habitus in lateral view

**Figure 2d.**
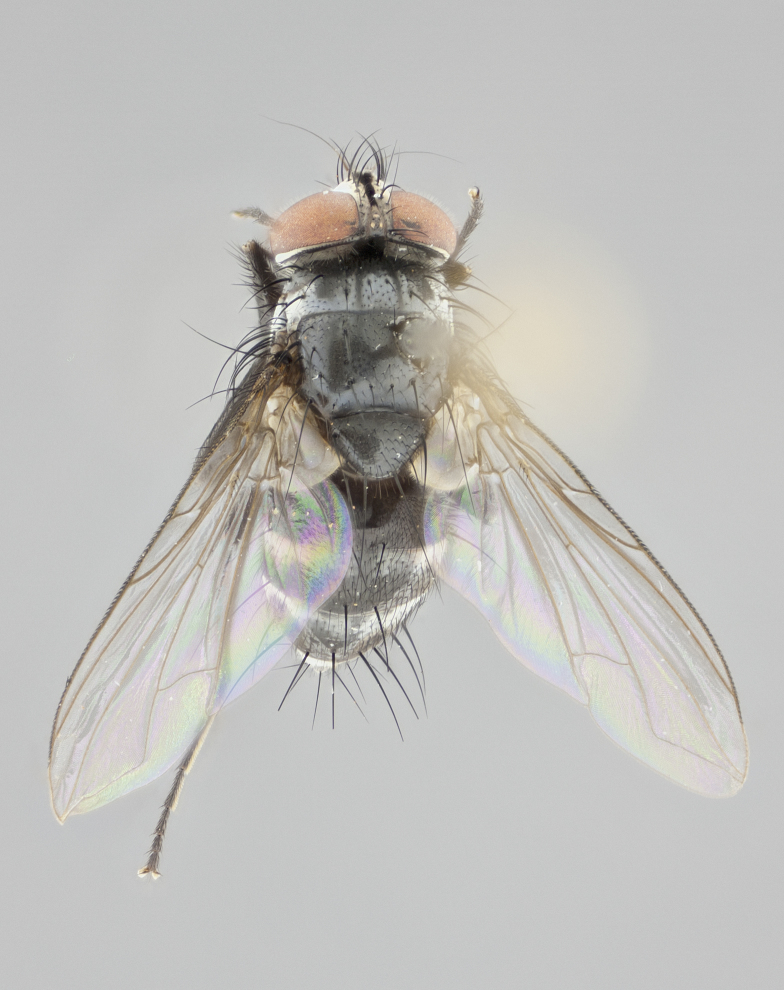
habitus in dorsal view

**Figure 2e.**
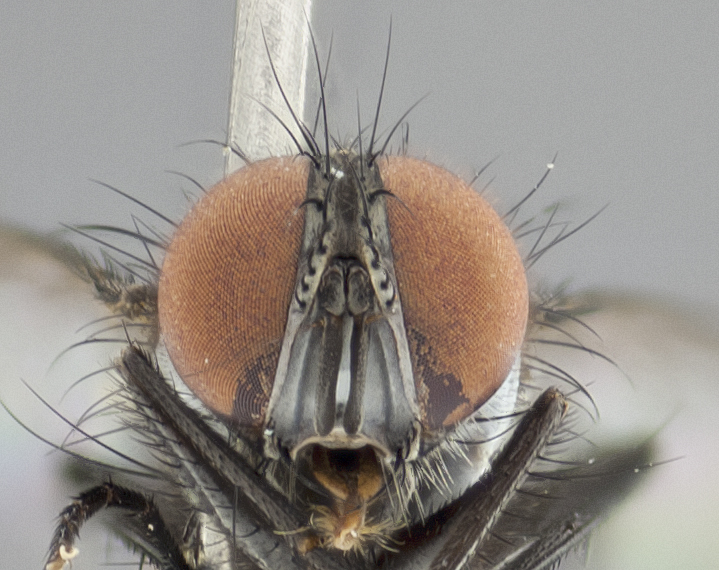
head in frontal view

**Figure 2f.**
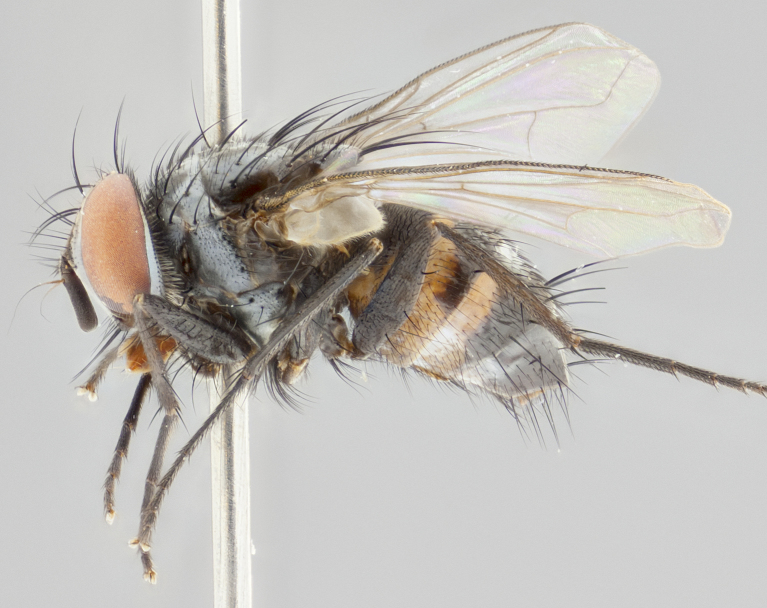
habitus in lateral view

**Figure 3a.**
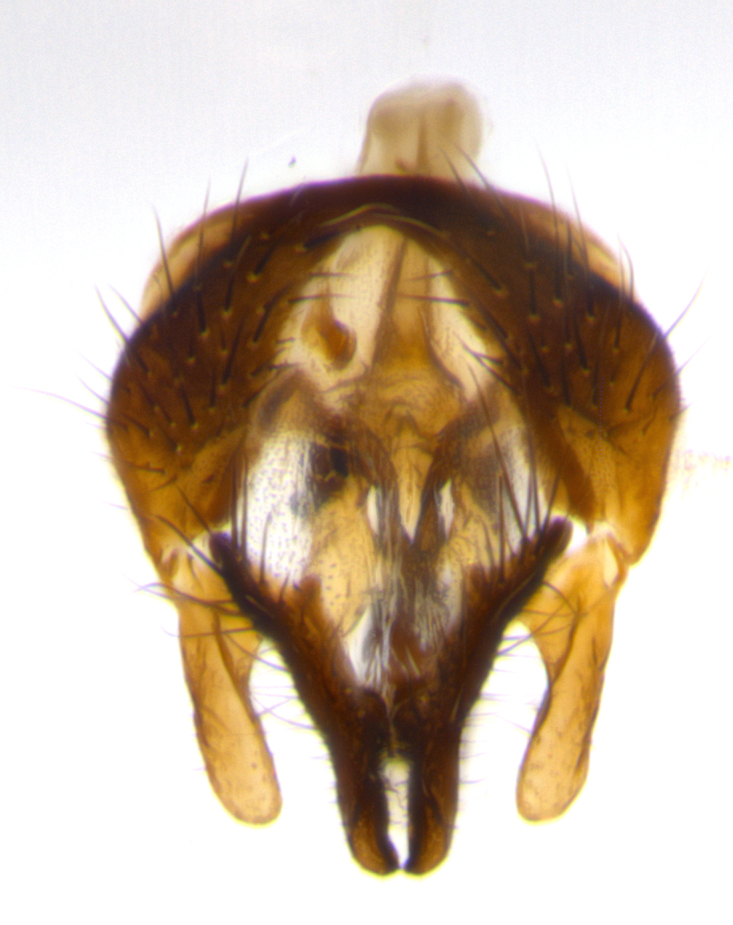
cerci and surstyli in dorsal view

**Figure 3b.**
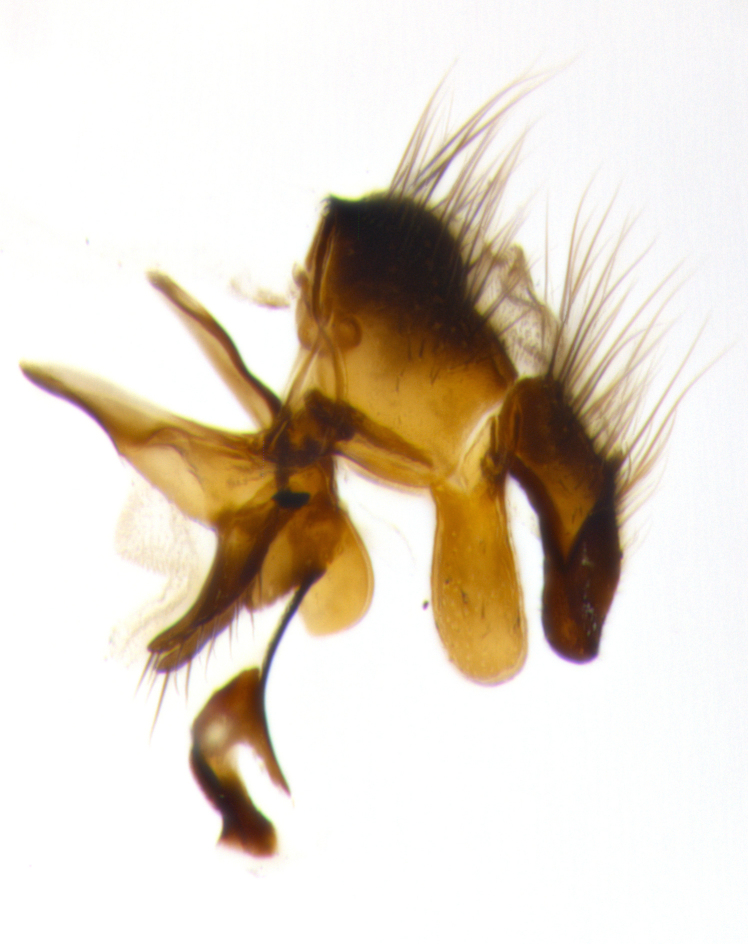
terminalia in lateral view

**Figure 3c.**
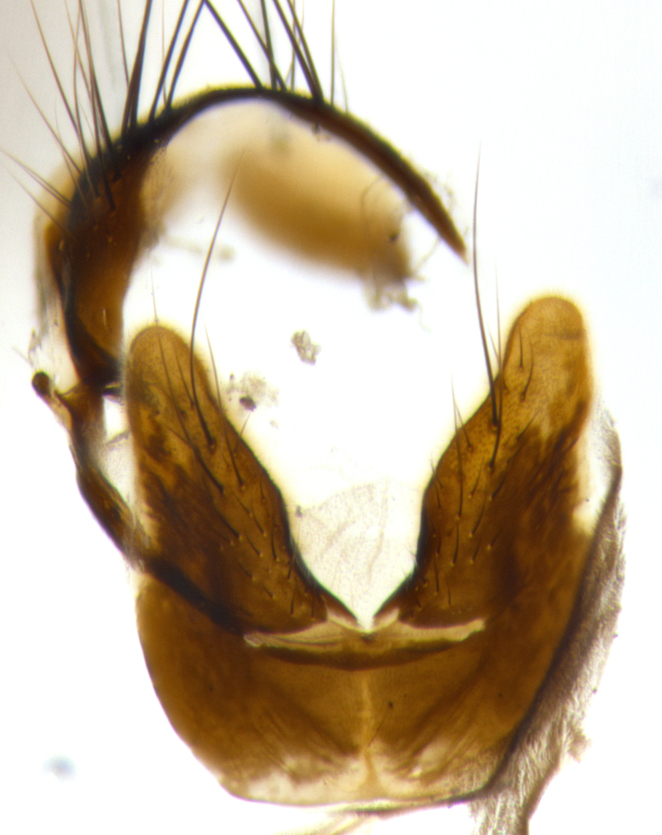
sternite 5 in ventral view

**Figure 4a.**
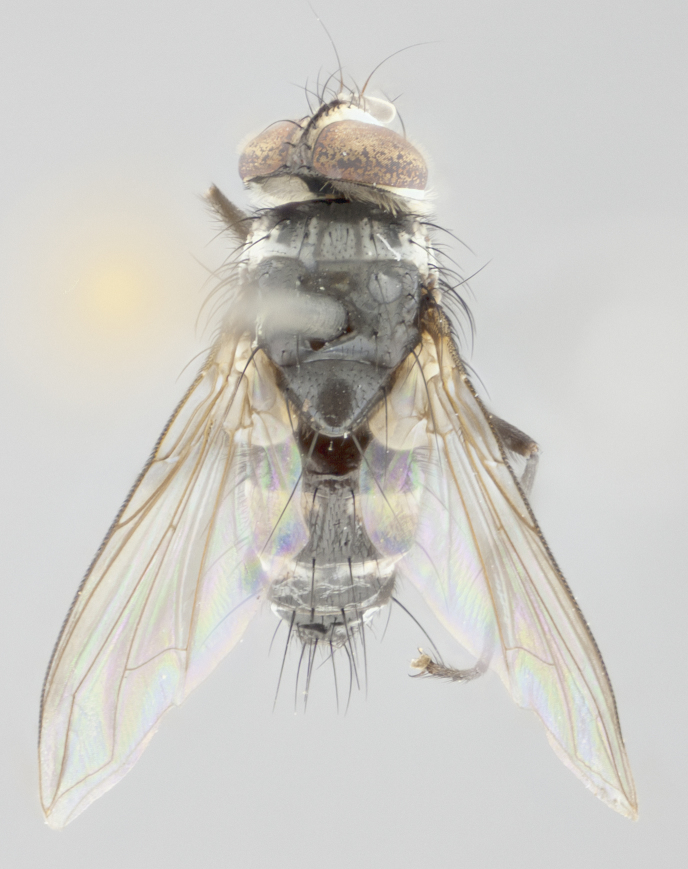
habitus in dorsal view

**Figure 4b.**
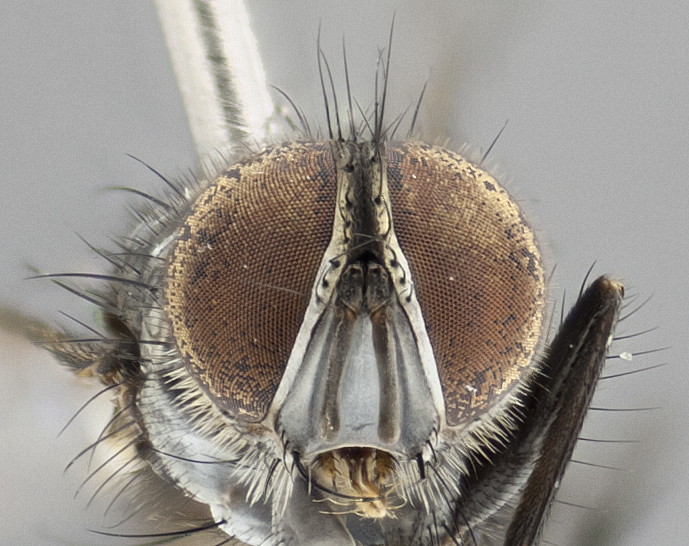
head in frontal view

**Figure 4c.**
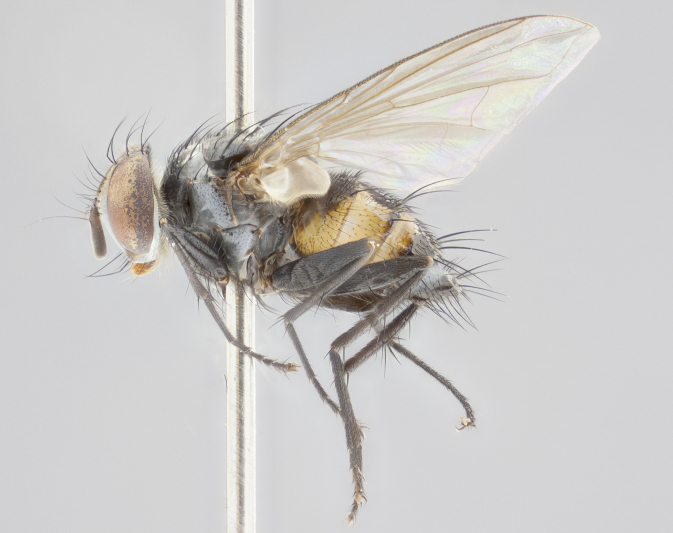
habitus in lateral view

**Figure 4d.**
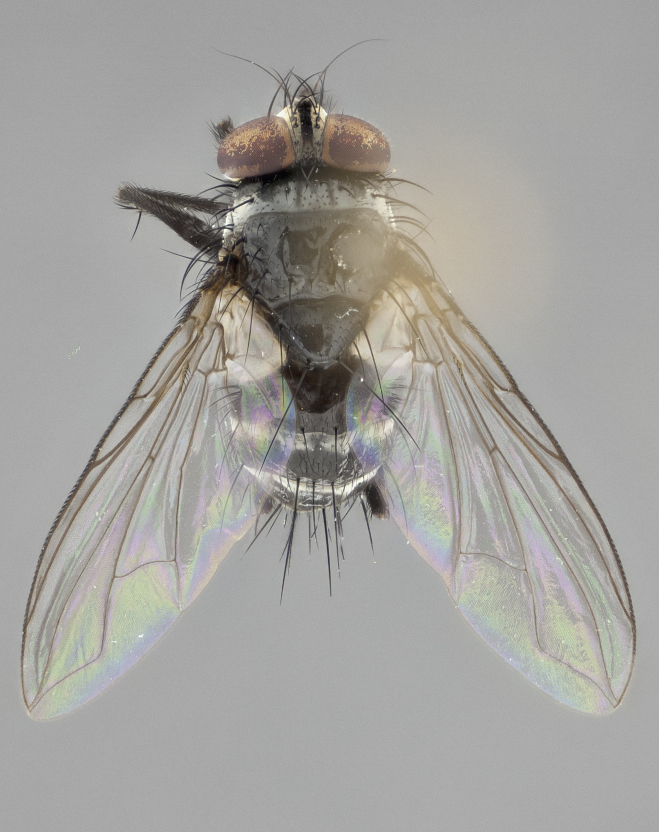
habitus in dorsal view

**Figure 4e.**
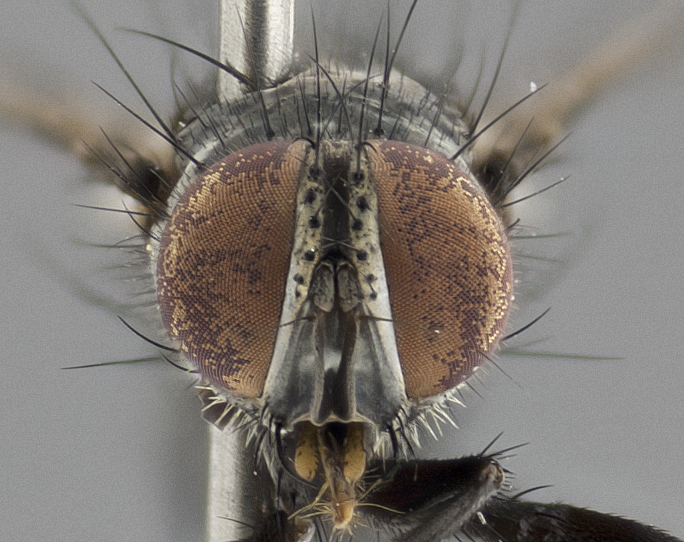
head in frontal view

**Figure 4f.**
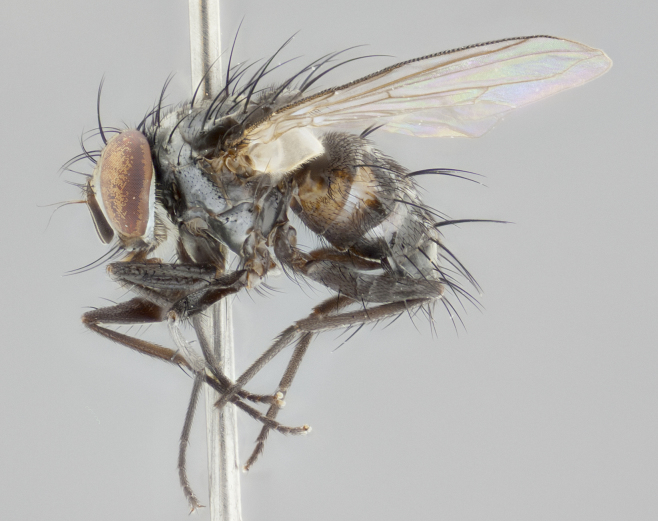
habitus in lateral view

**Figure 5a.**
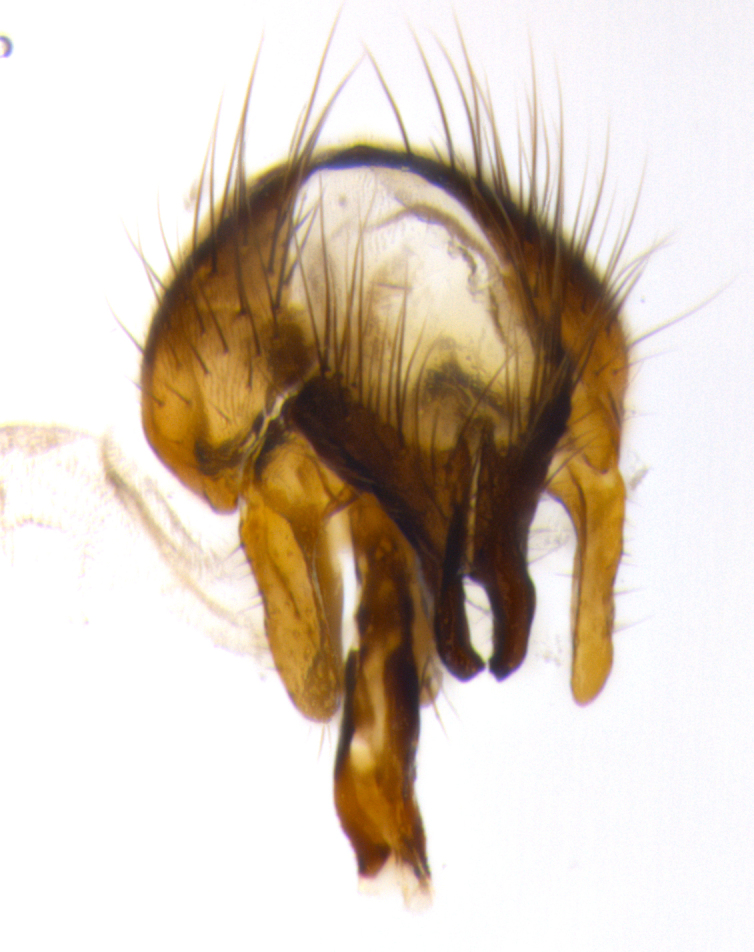
terminalia in dorsal view

**Figure 5b.**
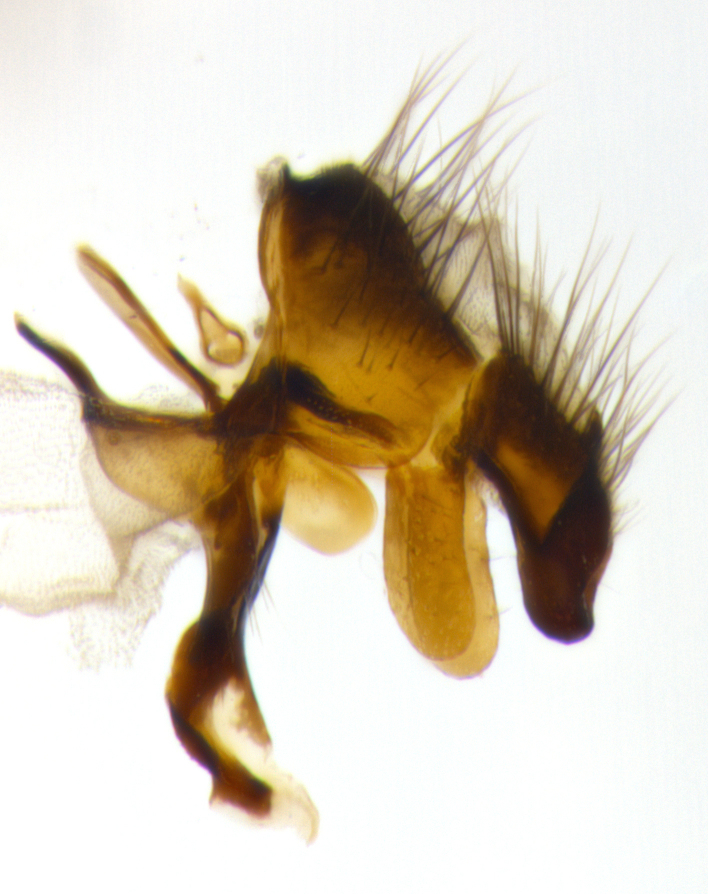
terminalia in lateral view

**Figure 5c.**
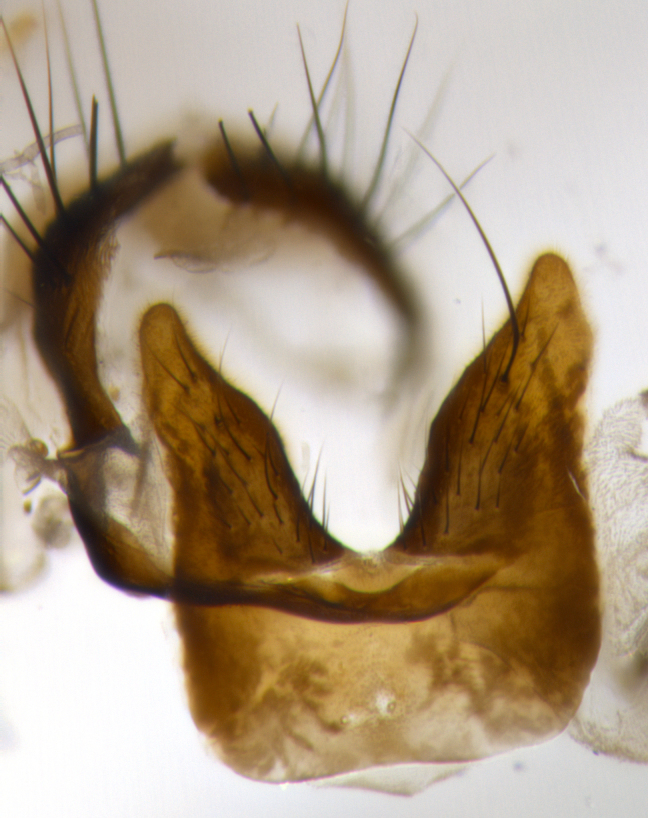
sternite 5 in ventral view
