# Supplementary material for: Two new species of Erythromelana Townsend, 1919 (Diptera: Tachinidae) from Area de Conservación Guanacaste in northwestern Costa Rica
Source: Biodivers Data J. 2016 Apr 19;(4):e7386. doi: 10.3897/BDJ.4.e7386 (PMC4867687; doi:10.3897/BDJ.4.e7386)
Supplement: Supplementary material 1 — NJ Tree Erythromelana (ACG) [file biodiversity_data_journal-4-e7386-s001.pdf]

BOLD TaxonID Tree

Title : SEARCH: Tax(Erythromelana), Geo(Costa Rica) [SEARCH1]
Date : 4-November-2015
Data Type : Nucleotide
Distance Model : Kimura 2 Parameter
Marker : COI-5P
Codon Positions : 1st, 2nd, 3rd
Labels : Extra Info, SampleID, Sequence Length
Filters : Length > 200
Colorization : [blue]=Stop Codons [red]=Contamination or misidentification

Sequence Count : 12
Species count : 2
Genus count : 1
Family count : 1
Unidentified : 0

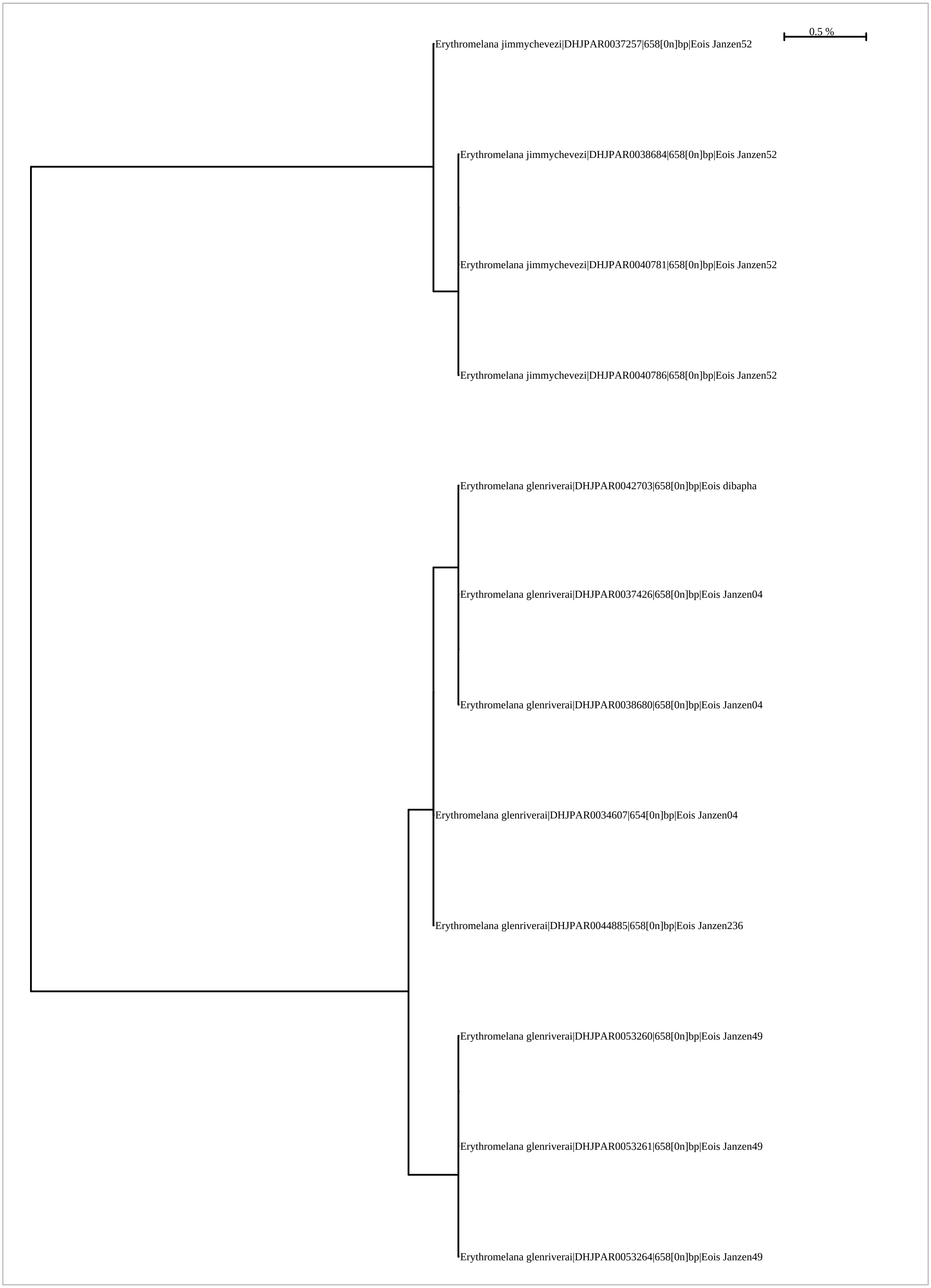
0.5 %
Erythromelana jimmychevezi|DHJPAR0037257|658[0n]bp|Eois Janzen52
Erythromelana jimmychevezi|DHJPAR0038684|658[0n]bp|Eois Janzen52
Erythromelana jimmychevezi|DHJPAR0040781|658[0n]bp|Eois Janzen52
Erythromelana jimmychevezi|DHJPAR0040786|658[0n]bp|Eois Janzen52
Erythromelana glenriverai|DHJPAR0042703|658[0n]bp|Eois dibapha
Erythromelana glenriverai|DHJPAR0037426|658[0n]bp|Eois Janzen04
Erythromelana glenriverai|DHJPAR0038680|658[0n]bp|Eois Janzen04
Erythromelana glenriverai|DHJPAR0034607|654[0n]bp|Eois Janzen04
Erythromelana glenriverai|DHJPAR0044885|658[0n]bp|Eois Janzen236
Erythromelana glenriverai|DHJPAR0053260|658[0n]bp|Eois Janzen49
Erythromelana glenriverai|DHJPAR0053261|658[0n]bp|Eois Janzen49
Erythromelana glenriverai|DHJPAR0053264|658[0n]bp|Eois Janzen49
